# Supplementary figures and images for: Rhythm and groove as cognitive mechanisms of dance intervention in Parkinson’s disease
Source: PLoS One. 2021 May 6;16(5):e0249933. doi: 10.1371/journal.pone.0249933 (PMC8101757; doi:10.1371/journal.pone.0249933)

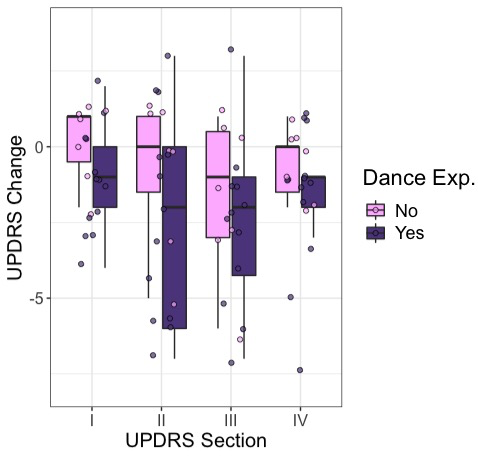

Supplement: S1 Fig — Tukey’s HSD post-hoc testing revealed no significant differences between changes in scores by UPDRS section (all p values > 0.05), nor were there significant differences by section for participants with and without DE (all p values > 0.1). (TIFF) [file pone.0249933.s001.tiff]
