# Supplementary material for: Rhythm and groove as cognitive mechanisms of dance intervention in Parkinson’s disease
Source: PLoS One. 2021 May 6;16(5):e0249933. doi: 10.1371/journal.pone.0249933 (PMC8101757; doi:10.1371/journal.pone.0249933)
Supplement: S1 Table — Each stimulus was designated as either “on the beat” or “off the beat”. (DOCX) [file pone.0249933.s002.docx]

| **Iversen and Patel Stimulus** | **On/Off the Beat** |
| --- | --- |
| **HTH_B0_v2** | **On** |
| **OWA_B-20_v2** | **Off** |
| **HSG_B20_v2** | **Off** |
| **KPS_B0_v2** | **On** |
| **NYN_B0_v2** | **On** |
| **ACL_B20_v2** | **Off** |
| **TJU_B20_v2** | **Off** |
| **SMA_B0_v2** | **On** |
| **SAS_B-20_v2** | **Off** |
| **RRW_B20_v2** | **Off** |
| **OCJ_B0_v2** | **On** |
| **PAN_B-20_v2** | **Off** |
