# Supplementary material for: Rhythm and groove as cognitive mechanisms of dance intervention in Parkinson’s disease
Source: PLoS One. 2021 May 6;16(5):e0249933. doi: 10.1371/journal.pone.0249933 (PMC8101757; doi:10.1371/journal.pone.0249933)
Supplement: S2 Table — Each excerpt has a unique groove rating as assigned by Janata, et al. [41]. The higher four groove ratings were designated as high-groove and the lower four as low-groove. (DOCX) [file pone.0249933.s003.docx]

| **Song Title** | **Artist** | **Genre** | **Groove Rating** | **Groove Designation** |
| --- | --- | --- | --- | --- |
| **Superstition** | **Stevie Wonder** | **Soul** | **108.7** | **High-Groove** |
| **Sing Sing Sing** | **Benny Goodman and His Orchestra** | **Jazz** | **97.4** | **High-Groove** |
| **In the Mood** | **Glenn Miller and His Orchestra** | **Jazz** | **96.9** | **High-Groove** |
| **Cheek to Cheek** | **Frank Sinatra** | **Jazz** | **85.7** | **High-Groove** |
| **What a Wonderful World** | **Louis Armstrong** | **Jazz** | **66.4** | **Low-Groove** |
| **’Til There was You** | **Etta Jones** | **Jazz** | **50.2** | **Low-Groove** |
| **Carolina in my Mind** | **James Taylor** | **Rock** | **49.0** | **Low-Groove** |
| **Comfortably Numb** | **Pink Floyd** | **Rock** | **42.3** | **Low-Groove** |
