# Supplementary material for: Rhythm and groove as cognitive mechanisms of dance intervention in Parkinson’s disease
Source: PLoS One. 2021 May 6;16(5):e0249933. doi: 10.1371/journal.pone.0249933 (PMC8101757; doi:10.1371/journal.pone.0249933)
Supplement: Attachment — Submitted filename: response_letter.docx [file pone.0249933.s004.docx]

# Reviewer #1:

1. Many more details about the dance classes should be provided in the Methods section. Currently, all I could find is participants were recruited from various dance classes around the United States.

*We thank the reviewer for pointing this out and we welcome the opportunity to expand on the details of the dance classes. All dance classes were taught by instructors who had received teaching certification from Dance for PD®. While the specific content of each class changed weekly, the structure was consistent and the primary form of movement was in a contemporary/modern style. Each class lasted 75 minutes and was set to live music. About 40 minutes were spent seated. While seated, instructors led exercises emphasizing muscle and limb extension and rhythmic movement. These exercises were typically broken into combinations focused on coordination (dancers might be instructed to clap on certain counts, or to alternate clapping and stomping), footwork (involving leg extension, flexing and pointing the feet, and raising heels to a forced arch position, for example), and torso (focusing on leaning forward and side to side, stretching the sides of the body, and coordinating arm movements with other motions), and explore a variety of musical tempos and rhythms. For the remainder of the class, instructors invited participants to stand if they were able. For about twenty minutes, participants performed exercises standing at the barre or holding on to the back of their chairs. These combinations emphasized plié, or bending and straightening the knees; lifting the heels off the ground to balance; and extending the legs and feet in different directions. Finally, participants moved across the floor for the last fifteen minutes of class, performing exercises involving weight shifting, marching, partnering (participants mirror others’ movement), and improvisation. In preparation for each combination, the instructor demonstrated a sequence of movements and then provided spoken cues while all participants performed the combination to music. Each class had at least one volunteer who provided dancers with support and assistance.*

*The above information is now included in Methods in a section titled “2.1.2 Intervention.”*

2. The control group has a lot more dance experience than those with PD. Why are the authors comparing these two groups?
   *Thank you for the opportunity to elaborate on our design, which included one session of testing non-PD control participants, as well as two sessions (pre- and post-intervention) of testing PD participants. While the tapping task with different levels of groove is a sensitive measure of rhythm and sensorimotor coupling, at present we know of no normed data from these assessments within the age range of those in our PD sample. Thus we believe it was necessary to validate these behavioral assessments against an age-matched control group without PD, within the scope of the present study.*

*Control participants were recruited from caretakers/close associates/family members, and thus were a sample of convenience that was age-matched to the PD group. 66.7% of PD participants and 63.1% of control participants had received at least one year of formal dance training. The reviewer is correct that the average years of dance training for control participants was about two times that of PD participants. However, neither PD nor control subjects’ durations of DE are normally distributed [see figure below for histogram of Years of DE by Group (PD and Control)]. Here, we simply categorized participants by the presence/absence of DE to assess the impact of DE on participants’ performance on objective rhythm tests.*

*Histogram of Years of DE for PD and Control groups.*

*In investigating the impact of discrepancies in years of DE on tapping task results, though, we did find associations between the number of years of dance experience and Shannon entropy for some participants. We found a negative correlation between post-intervention PD participants’ tapping task entropy and years of dance experience (Pearson’s correlation: r = -0.248, p = 0.011*). However, correlations between both pre-intervention PD entropy values and years of DE (r = -0.150, p = 0.0648) and control participants’ entropy values and years of DE (r = -0.190, p = 0.0640) did not reach significance.*

*To tease apart the effect of Years of DE from the effect of group (PD vs. control) on tapping entropy, we included “Years of DE” as a fixed effect in all LME models comparing control and PD Shannon entropy. For LMEs comparing pre-intervention PD and control log(Entropy) values, likelihood ratio tests revealed that adding Group, χ^2^(1) = 7.51, p = 0.00612** and Groove, χ^2^(1) = 9.48, p = 0.00208** significantly and independently improved model fit. Adding Years of DE, χ^2^(11) = 17.1, p = 0.104, did not improve model fit. F-tests confirmed the main effects of Group [F(1,47) = 7.79, p = 0.00757**] and Groove [F(1,342) = 9.58, p = 0.00213**], indicating that pre-intervention PD log(Entropy) values were significantly higher than control values, and again that high-groove entropy values were lower than low-groove values. All interaction terms were non-significant (all p values > 0.2).*

*LMEs comparing post-intervention PD and control log(CV) values revealed that including Groove, χ^2^(1) = 12.6, p = 0.000393***, but not Group, χ^2^(1) = 1.88, p = 0.17, significantly improved model fit. F-tests revealed a significant main effect of Groove [F(1,279) = 12.8, p = 0.000408***], indicating again that high-groove entropy values were lower than low groove values. While including Years of DE improved model fit, χ^2^(11) = 22.2, p = 0.0228*, an F-test failed to reject the null hypothesis that Years of DE had no independent effect on log(Entropy) [F(1,11) = 1.89, p = 0.0853]. All interaction terms were non-significant (all p values > 0.2). Together, these results demonstrate that PD participants reduced their tapping variability after the intervention, with tapping entropy more similar to that of controls. Furthermore, while tapping entropy varied across songs, high-groove entropy was consistently lower than low-groove entropy, and duration of DE did not interact with these effects.*

*These updated LME models above, with Years of DE as a fixed effect, have been incorporated into Section 3.3 of our manuscript. We have also noted in the discussion that with a larger sample size, further investigation may reveal relationships between tapping task performance and duration of DE that are not captured by the current study. While we thank the reviewer for pointing out that the controls had more years of DE on average than our PD group, we hope these new results assuage any potential concerns of the between-group effects being confounded by Years of DE.*

3. The hit rates in the table could use some metric for units. Same for tapping task, high groove and low groove. I understand if it's 'difficult' to produce a unit, but try to explain what is meant by the score.

*We thank the reviewer for pointing this out and agree that more explication would help clarify the data. In our original manuscript, we expressed hit rate as the proportion of correctly detected differences given the overall number of differences, whereas false alarm rate was the proportion of trials where the response was “different” given that the correct response was “same.” In this revision, we express these values as percentages.*

*For the tapping task, we have also clarified that the values listed for the tapping task reflect Shannon entropy of phase angles. While we understand the reviewer’s ask for units for entropy, some sources cite “Shannon” as the unit but in this case that unit is less appropriate because it applies to probabilities of phase angles, rather than a more classical linear measure. Since entropy is by nature a proportional value, in this revision we explain these values by giving a more in-depth conceptualization of these measures. We also provide the theoretical upper and lower bounds of entropy for reference. In section 2.3.3, we now clarify that Shannon entropy reaches zero if the probability of a certain IOI value (or phase angle) is 1. This gives us the possible range of entropy values: because we used 400 bins to construct polar histograms of each tapping trial, the highest possible entropy is -(400*1/400*log(1/400) = 2.602, indicating complete randomness, while the lowest is 0, indicating robotic tapping.*

4. The UPDRS III scores indicate these individuals had almost no parkinson's- these are Hoehn and Yahr stage 1 patients. Really? If so, the complications of therapy scores are curious. I would not expect patients with such low scores on the UPDRS III to have much if any medication complications.

*The reviewer is correct that these patients mostly fall in H&Y Stage 1. However,*

*we note that the UPDRS IV scores are very low across the board. In a study assessing the validity of UPDRS Section IV, Raciti et al. (2016), found that the average UPDRS IV score for PD patients who did not experience motor fluctuations--and thus experienced few, if any, medication complications--was 2.87 ± 2.4. For patients experiencing motor fluctuations, the UPDRS IV scores averaged 5.7 ± 2.6. . Our data thus converge with the reviewer’s expectation that low UPDRS III scores would beget few, if any, complications of therapy. Out of a possible 23 points in UPDRS IV, the average score for pre-intervention participants in our study was 3.8 ± 3.84, and post-intervention, 3.05 ± 3.09. These data reflect a participant pool in which most, but not all PD participants experience very few medication complications, consistent with the reviewer’s expectation.*

*Raciti, L., Nicoletti, A., Mostile, G., Bonomo, R., Contrafatto, D., Dibilio, V., . . . Zappia, M. (2016). Validation of the UPDRS section IV for detection of motor fluctuations in Parkinson's disease. Parkinsonism Relat Disord, 27, 98-101. doi:10.1016/j.parkreldis.2016.03.008*

5. Table 1 should be divided into subject characteristics and then outcome variables- make two tables. It is confusing too- the outcome variables initial and follow up- does that mean they are presenting overall means for both groups? Or means for just the PD group? Seems like at least two columns are missing in this table.

*Table 1 has been separated into two tables as indicated. We have also clarified that there are three columns for the outcome variables table: PD pre-intervention, PD post-intervention, and control values. Control participants do not have pre- and post-intervention values, so are listed in a single column.*

6. Figure 2 title- consider rewording- "separated by..." does not make a lot of sense.

*We agree that the initial wording was confusing. We have now updated the text to read, “With and without music experience (ME) and dance experience (DE)” instead of “separated by…”*

7. Section 3.3 goes on to include a lot of statistical methods that should be placed in Methods/Statistical analysis section. Overall - this section is written up well in terms of statistical precision- but please remove anything that is methods and try to be more concise if possible.

*Thank you for pointing this out. We have removed all description of LME model construction from Section 3.3 and have included it in Section 2.3.3 (Methods: Statistical analysis). Section 3.3 is now more concise.*

8. The argument that low and high groove represents "sensorimotor coupling ability" needs to be strengthened in the introduction.

*We agree with the reviewer that the introduction could use more explanation on the role of groove in sensorimotor coupling. Evidence for the role of groove in sensorimotor coupling comes from results involving transcranial magnetic stimulation (TMS) of the motor cortex: when asked to tap along to different songs, healthy participants show the most spontaneous motor excitability, as indicated by motor evoked potentials in response to TMS, in response to high-groove songs as compared with songs categorized as low-groove. This suggests that groove mediates the way auditory rhythms excite the motor system; in other words, groove drives sensorimotor coupling. This is now added to the Introduction.*

9. Maybe a figure of the concepts- of what tapping is supposed to represent, of what the BAT is supposed to represent or what entropy is supposed to represent would help the reader make their way through the results description.

*We agree with the reviewer that a visual / conceptual overview would be helpful. In this revision we add a new Figure 1 to show our conceptualizations of the problem of dance experience and intervention, the cognitive constructs and neural systems that they tap into, and the tools that we use to assess these constructs. We thank the reviewer for this suggestion and we hope that it clarifies our thinking on dance for PD.*

***Fig 1****. Conceptual overview of relationships between relevant cognitive and behavioral assessments, constructs, and activities in the context of Parkinson’s disease.*

10. We know the patients took weekly classes for four months- please give more detail. How many classes did the patients actually take, ie percent adherence, or number of classes taken out of total offered, etc. Summary data would be fine.

*In addition to what was said in response to Point 1, we now include additional information on how compliance with the intervention affected inclusion/exclusion criteria. The minimum requirement for dance class attendance, then, was one class per week, or 16 total classes. If by the post-intervention meeting (four months after the initial meeting) participants had failed to attend at least one class per week, their data were excluded from follow-up analysis. We did not exclude participants for additional class attendance, or for participating in any other interventions. We have now included clarification of the 16 class requirement (one per week) in Section 2.1.4.*

11. The measures (BAT, tapping task) themselves are very interesting and likely of use to those who study dance and music. The study design and lack of clear role of the control group is a detractor to the paper. If the authors could address these issues, this would strengthen the paper.

*Thank you for your comment. We have now added more of a conceptual overview, including by adding a new Figure 1. We refer to the BAT and tapping task, along with other behavioral and cognitive components of our study, in the introduction and in Figure 1. We now also add a rationale for use of the control group. To validate the relatively novel use of these tasks to assess rhythm capabilities and sensorimotor coupling, we needed an additional control group who had no PD to serve as a comparisonator for PD participants’ performance on objective rhythm tasks at both timepoints*

# Reviewer #2:

1. There is no clear description of the nature of the dance intervention, i.e. the content of the dance classes. The study is very clear in reporting the tests performed pre- and post-intervention, but mentions nothing in the methods section about the intervention itself. What kind of dance classes were these? There are many different ways to run a dance class for people with PD. There is folk dance, tango (see work by Hackney and colleagues) and Dance for PD which is widely used in many countries. The authors mention three sites in different parts of the US in which they collected data. Did all three sites run the same intervention, or did each site run different classes? Were these classes coordinated/controlled by the experimenters, in terms of their content and methods? Finally, how many classes did participants attend on average? If we don’t know what these classes were, and if we don’t know if they all did the same thing it is very hard to interpret these results.

*All dance classes were taught by instructors who had received teaching certification from Dance for PD®. While the specific content of each class changed weekly, the structure was consistent and the primary form of movement was in a contemporary/modern style. Each class lasted 75 minutes and was set to live music. About 40 minutes were spent seated. While seated, instructors led exercises emphasizing muscle and limb extension and rhythmic movement. These exercises were typically broken into combinations focused on coordination (dancers might be instructed to clap on certain counts, or to alternate clapping and stomping), footwork (involving leg extension, flexing and pointing the feet, and raising heels to a forced arch position, for example), and torso (focusing on leaning forward and side to side, stretching the sides of the body, and coordinating arm movements with other motions), and explore a variety of musical tempos and rhythms. For the remainder of the class, instructors invited participants to stand if they were able. For about twenty minutes, participants performed exercises standing at the barre or holding on to the back of their chairs. These combinations emphasized plié, or bending and straightening the knees; lifting the heels off the ground to balance; and extending the legs and feet in different directions. Finally, participants moved across the floor for the last fifteen minutes of class, performing exercises involving weight shifting, marching, partnering (participants mirror others’ movement), and improvisation. In preparation for each combination, the instructor demonstrated a sequence of movements and then provided spoken cues while all participants performed the combination to music. Each class had at least one volunteer who provided dancers with support and assistance.*

*In addition to what was said in response to Point 1, here is some additional information on how the compliance with the intervention affected inclusion/exclusion criteria. If by the post-intervention meeting (four months after the initial meeting) participants had failed to attend at least one class per week, their data were excluded from follow-up analysis. The minimum requirement for dance class attendance, then, was one class per week, or 16 total classes. We did not control for additional class attendance or other interventions, nor did we collect that information.*

*This information is now included in Methods in a section titled “2.1.2 Intervention,” as well as in a response to Reviewer 1’s Points 1 and 10.*

2. Another issue is that half of the participants who finished the intervention had been participating in dance classes for some time before the pre-test. That means that this is not a real pre-test, in the sense that you are not measuring what happened before the intervention, the intervention was going on before the pre-test as well. However, what is puzzling is that one would expect greater effects of the intervention at the UPDRS for people with no dance experience but instead the opposite was found to be the case. The authors could discuss this in the discussion section.

*We acknowledge that the pre-test did not represent a true baseline in the sense that all PD participants in the study had been previously attending PD dance classes. However, we believe that the four month period does capture some contrast against an earlier stage in participants’ experience with PD dance classes, as demonstrated by the fact that PD participants in our study exhibited symptom improvement, as well as the finding that participants with dance experience exhibited greater improvements. These results point to a need for further investigation into the neuroprotective effects of dance early in life. As you mention, this would ideally be done at any earlier point in disease onset in order to establish a true baseline. Our research suggests that dance experience makes you more susceptible to benefits of dance intervention later on, which is a point that we hope is addressed with future research.*

3. There are other, more minor methodological issues with the study, but I mention them as minor because the authors acknowledge them in the discussion as limitations, for example issues with the control group and the drop in the number of participants post-test. These, and other of the limitations are part and parcel of this type of research which is extremely hard to perform. However, the content of the classes and its consistency across sites is a major issue.

*We appreciate your acknowledgment of the challenges inherent to this kind of research and hope that we have sufficiently addressed your comments about the content of the classes and their consistency.*

4. There are two recent reviews on the topic that the authors could include, one by Bek et al (2020), NNR; and one by Carapellotti (2020) in PLoS one.

*Thank you for bringing our attention to these reviews. We have now included reference to both reviews in the introduction, and to Carapellotti et al., in the discussion.*
