# Supplementary material for: Rhythm and groove as cognitive mechanisms of dance intervention in Parkinson’s disease
Source: PLoS One. 2021 May 6;16(5):e0249933. doi: 10.1371/journal.pone.0249933 (PMC8101757; doi:10.1371/journal.pone.0249933)
Supplement: Attachment — Submitted filename: PD Response Letter PLoS One 2.docx [file pone.0249933.s005.docx]

Dear Dr. McKay,

Thank you for handling our manuscript, “Assessing and Predicting Efficacy of Dance Intervention for Parkinson’s Disease,” for possible publication in *PLoS ONE.* We also thank you and the reviewers for careful feedback and helpful comments. We now submit a revised version, with a title change, additions to the description of data analysis in R, and additions to the limitations section. Point-by-point responses to the reviewers’ comments are below. We hope that you might find this version to be suitable for publication for the wide readership of *PLoS ONE*.

Sincerely,

Psyche Loui

Anna Krotinger

# Academic Editor:

1. Due to the sophistication of the analytic methods used, please provide the specific R function used to fit linear mixed models (stats::glm, LmerTest::lme4, etc.), the function used to perform likelihood ratio tests (anova, drop1(lm1, test="F")), and whether maximum likelihood (ML) or restricted maximum likelihood (REML) was used for model fitting. Without these details readers are unable to critically evaluate results, which can vary somewhat depending on the values chosen.

*Thank you for pointing this out! We have updated section 2.3.3 to read:*

*“Linear mixed-effects models (LMEs) were constructed using the R function LmerTest::lme4 to investigate whether Groove (High vs. Low), Group (PD vs. Control), and their interaction affected IOI values. First, a base model was constructed using IOI as the dependent variable and participant ID as a random effect. Then, Groove and Group were added as fixed effects. Maximum likelihood (ML) was used for model fitting. Models were compared via likelihood ratio tests using the anova function. All code for analyses and figures are freely available on https://doi.org/10.6084/m9.figshare.13034165.v1.”*

# Reviewer #1:

1. I have a suggestion that I think is pretty important- change the title to something more about music, groove and/or rhythm. This aspect is by far the most interesting aspect of this paper. You can also include "Dance for PD, a contemporary dance style" or "contemporary dance in the title rather than simply "dance". I am not sure about 'efficacy' either.

also- we need to see what the editor thinks but the design of the project is important too, given this is not a RCT or a pilot study- it's more a convenience sample, or maybe something "quasi -experimental". I think including that in the title or somewhere in the abstract and methods is important.

*We thank the reviewer for pointing out that the title could be improved by including reference to groove and rhythm. We have altered it to read:*

*“Rhythm and Groove as Cognitive Mechanisms of Dance Intervention in Parkinson’s Disease”*

*We hesitate to specify the dance intervention as exclusively “contemporary” in the title of the study. In our description of the intervention in section 2.1.2, we describe the intervention thus: “While the specific content of each class changed weekly, the structure was consistent and the primary form of movement was in a contemporary/modern style.” Had the movement in every class been* exclusively *contemporary, we would feel comfortable noting this in the title. However, each teacher has license to draw influence from other styles of dance, so even if the majority of combinations in a given class are in a contemporary style, there might be a single piece of choreography that draws on African or Latin dance styles, for example. We have clarified this in the body of the manuscript (2.1.2):*

*“While the specific content of each class changed weekly and teachers were free to draw on different dance styles (e.g. African or Latin) while planning choreography, the structure was consistent and the primary form of movement was in a contemporary/modern style.”*

# **Reviewer #2**:

1. Please specify in the limitations that the results may not generalize to a study population without ongoing dance training at baseline, and that you would either expect increased or decreased efficacy in such a population.

*We agree with the reviewer that results may differ if dance training had not been ongoing at baseline. We have now added to the limitations section of Discussion to read:*

*“Another caveat of these findings is the lack of random assignment and a control intervention: due to limitations in time and resources, we were only able to recruit participants who self-selected into dance intervention, and we were not able to compare dance intervention against a control intervention in this study. Although we had a well-matched control group who were not affected by PD, this control group did not receive intervention, and was smaller in sample size than the PD group. Nevertheless, our healthy control group provided aged-matched control data for the BAT and the rhythmic tapping tasks, which showed better performance (superior beat perception and lower tapping variability) than the pre-intervention but not the post-intervention PD participants.*

*Furthermore, all PD participants had already been attending Dance for PD classes prior to enrolling in our study. Thus, our results may not generalize to a population without ongoing dance training at baseline. Future studies should include assessments prior to the start of the intervention in order to characterize the trajectory of possible improvements due to dance intervention.”*
